# A Depth Awareness and Learnable Feature Fusion Network for Enhanced Geometric Perception in Semantic Correspondence

**DOI:** 10.3390/s24206680

**Published:** 2024-10-17

**Authors:** Fazeng Li, Chunlong Zou, Juntong Yun, Li Huang, Ying Liu, Bo Tao, Yuanmin Xie

**Affiliations:** 1 Key Laboratory of Metallurgical Equipment and Control Technology of Ministry of Education, Wuhan University of Science and Technology, Wuhan 430081, China; xieyuanmin@wust.edu.cn; 2 College of Mechanical Engineering, Hubei University of Automotive Technology, Shiyan 442000, China; zouchunlongh_hapm@163.com; 3 Research Center for Biomimetic Robot and Intelligent Measurement and Control, Wuhan University of Science and Technology, Wuhan 430081, China; 4 Hubei Province Key Laboratory of Intelligent Information Processing and Real-Time Industrial System, Wuhan University of Science and Technology, Wuhan 430081, China; huangli82@wust.edu.cn; 5 College of Computer Science and Technology, Wuhan University of Science and Technology, Wuhan 430081, China; 6 School of Mechanical Engineering, Hubei Engineering University, Xiaogan 432000, China; liuying3025@wust.edu.cn; 7 Hubei Key Laboratory of Mechanical Transmission and Manufacturing Engineering, Wuhan University of Science and Technology, Wuhan 430081, China

**Keywords:** semantic correspondence, depth awareness, feature fusion, learnable weights, contrastive learning

## Abstract

Deep learning is becoming the most widely used technology for multi-sensor data fusion. Semantic correspondence has recently emerged as a foundational task, enabling a range of downstream applications, such as style or appearance transfer, robot manipulation, and pose estimation, through its ability to provide robust correspondence in RGB images with semantic information. However, current representations generated by self-supervised learning and generative models are often limited in their ability to capture and understand the geometric structure of objects, which is significant for matching the correct details in applications of semantic correspondence. Furthermore, efficiently fusing these two types of features presents an interesting challenge. Achieving harmonious integration of these features is crucial for improving the expressive power of models in various tasks. To tackle these issues, our key idea is to integrate depth information from depth estimation or depth sensors into feature maps and leverage learnable weights for feature fusion. First, depth information is used to model pixel-wise depth distributions, assigning relative depth weights to feature maps for perceiving an object’s structural information. Then, based on a contrastive learning optimization objective, a series of weights are optimized to leverage feature maps from self-supervised learning and generative models. Depth features are naturally embedded into feature maps, guiding the network to learn geometric structure information about objects and alleviating depth ambiguity issues. Experiments on the SPair-71K and AP-10K datasets show that the proposed method achieves scores of 81.8 and 83.3 on the percentage of correct keypoints (PCK) at the 0.1 level, respectively. Our approach not only demonstrates significant advantages in experimental results but also introduces the depth awareness module and a learnable feature fusion module, which enhances the understanding of object structures through depth information and fully utilizes features from various pre-trained models, offering new possibilities for the application of deep learning in RGB and depth data fusion technologies. We will also continue to focus on accelerating model inference and optimizing model lightweighting, enabling our model to operate at a faster speed.

## 1. Introduction

Semantic correspondence provides pixel-wise matching, even for visually dissimilar images, enabling stable and accurate correspondences based on semantic information. This underpins a variety of downstream tasks, such as style transfer [1,2], scene understanding [3,4,5], pose estimation [6,7], and 3D reconstruction [8,9], by establishing a solid application foundation. Traditional dense correspondence tasks typically involve geometric constraints [10,11]; for example, epipolar geometry, commonly used in robot simultaneous localization and mapping (SLAM) systems [12,13,14,15,16]. They often used feature detection and description algorithms, such as scale-invariant feature transform (SIFT) [17] and sped-up robust features (SURF) [18], to obtain descriptors and perform matching. During matching, geometric constraints were first used to eliminate obvious outliers, then optimization algorithms (e.g., RANSAC [19]) were employed to optimize the matches. However, geometric constraints are generally absent in semantic correspondence tasks. Semantic correspondence must address challenges, including significant intra-class appearance variations and potential non-rigid transformations when the constraints of epipolar geometry or affine transformation are lost.

To address these challenges, due to the tremendous advantages of deep convolutional neural networks (CNNs) in image classification tasks [20,21], a series of methods [22,23] were designed as processes to address the aforementioned challenges based on the stable feature extraction capability of CNNs, i.e., feature extraction, cost volume construction, and flow estimation or field regression. Methods such as [24,25,26,27] utilized CNNs for feature extraction, capable of learning relatively stable feature representations even with significant intra-class appearance variations, achieving more accurate correspondences.

In recent years, with the rapid development of the self-supervised learning paradigm [28,29], visual backbone models trained on large-scale datasets using self-supervised learning and generative models gradually emerged. Large-scale self-supervised learning (SSL) models, such as the DINO series [30,31], were pre-trained on large datasets using designed pretext tasks or contrastive learning to learn robust representations, which were applied to an increasing number of downstream tasks [32,33,34]. Generative models, such as stable diffusion (SD) [35], leverage the generative capabilities of diffusion models [36,37] to bring a promising future to applications such as text-to-image and image-to-image generation.

**Figure 1:**
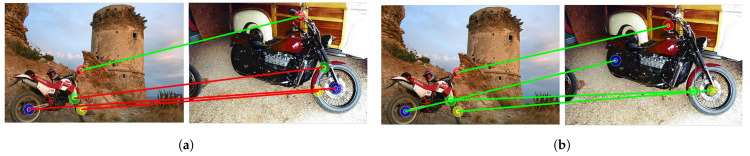
The previous work [39] (**a**) found it challenging to differentiate between the front and rear wheels of motorcycles, and our method (**b**) aids in alleviating this issue. Green lines represent correct matches, and red is incorrect.

To enhance feature extraction capabilities, some methods have already utilized features from SSL and generative models. For example, ref. [38] combined features from different time steps and various layers of SD into a descriptor. However, SD features provided more spatial messages but less semantic information. To improve the representation capability and generalizability of the descriptor, ref. [39] also integrated the most representative features from both DINO and SD using principal components analysis (PCA) and fixed weights. However, although the features of DINO possess strong generalizability and domain generalization performance, the practice of simply joining SD features and DINO features with a fixed weight and utilizing each part of the features equally may lead to insufficient effective feature weights and diminish the principal components of the features. Additionally, features learned by self-supervised models struggle to distinguish between similar independent parts with certain symmetries [40], such as the front and rear wheels of motorcycles, as shown in Figure 1. These parts have similar image details and semantic information, resulting in similar outputs for similar inputs in SSL methods. Furthermore, in 2D images, the depth information of objects is almost lost during the imaging process, making it difficult to perceive the geometric structures of objects.

In this paper, to address the insufficient perception of object geometric structures in existing methods, we propose a latent depth awareness module to enhance the model’s understanding of geometric structures, thereby mitigating depth ambiguity issues caused by RGB images. Inspired by [41,42], using depth features from depth estimation or depth sensors can provide more geometric information about objects, extending the features. Specifically, we estimate the relative depth for each feature map, divide it into discrete intervals, and construct a pixel-wise discrete depth distribution. We fuse this with SD and DINO features, weighting them by depth information, which alleviates the problem of depth ambiguity and improves the ability to understand the geometry of objects.

To address the issues of feature component ambiguity and the curse of dimensionality, we designed a feature fusion module to identify the optimal feature fusion strategy, ensuring that both types of features are effectively leveraged. First, we use a fusion block to align the channels of the various features. Then, we use learnable weights to fuse the features. By optimizing the learnable weights through contrastive learning, we ensure that each part of the features is fully utilized. Compared to previous methods, our approach alleviates the problem of depth ambiguity, optimizes the perception of object structures, and uses learnable weights to reasonably fuse various features, enabling each part of the features to maximize their effectiveness.

In the experiments (Section 4), our method improved the PCK@0.1 metric by approximately seven points on the SPair-71k dataset and by approximately nine points on the reconstructed AP-10K benchmark compared to previous methods, significantly enhancing matching accuracy.

To summarize, our main contributions are as follows:

- We introduce a novel latent depth awareness module leveraging monocular depth estimation to enhance the understanding of object geometry, providing richer structural information and addressing depth ambiguity.
- We design a feature fusion module with learnable weights, aligning and integrating features from DINO and stable diffusion layers, improving the utilization of effective features and mitigating the curse of dimensionality.
- We build a semantic correspondence matching system, improving the model’s understanding of the overall object structure and laying the foundation for more robust matching.

The remaining sections of this paper are organized as follows: we provide a brief overview of the development of existing semantic correspondence studies in Section 2. Section 3 offers a detailed description of the proposed latent depth-aware module and learnable weight-guided feature fusion module. The experimental results can be found in Section 4. Finally, Section 5 summarizes the main contents and discussions presented in this article.

## 2. Related Works

### 2.1. Semantic Correspondence

The semantic correspondence task originated from the complex image alignment task, which involves obtaining semantic-level feature correspondences for the same instance or different instances of the same category from different perspectives, positions, and scales. SIFT Flow [43] was the first to establish cross-scene image correspondences by utilizing the robust representation capabilities of SIFT [17]. The first step in the semantic correspondence pipeline is usually feature extraction. Traditional methods often rely on feature descriptors for representation [44,45], but these have certain limitations with respect to viewpoint changes. Due to the success demonstrated by convolutional neural networks in the field of computer vision [46], CNNs gradually replaced traditional methods as the commonly used general feature extractors to learn better representations of images [25,47,48]. They extract multi-layer features and construct multi-scale features for feature aggregation, laying the foundation for subsequent CNN-based semantic matching systems. Recent works have achieved excellent results by utilizing the self/cross-attention mechanisms of transformers for feature enhancement and aggregation [49,50,51]. Additionally, vision backbone models based on self-supervised learning or generative models [30,35] can directly discover robust features that sometimes surpass those of existing supervised learning paradigms, providing new solutions for semantic correspondence feature extraction. However, self-supervised learning models primarily learn invariant features, meaning that the same features maintain the same representations [40]. This is determined by the paradigm of self-supervised learning, where common self-supervised learning methods design various pretext tasks to learn more stable and general features. The represented features are also similar for parts of objects with similar functions. For example, the four wheels have similar representations in the case of car wheels, but it is difficult to distinguish between the front and rear wheels.

After obtaining image features, the similarity between the pixels of two images is usually determined by calculating the cost volume of the feature maps [52]. Some works manually designed the cost volume calculation [25,53], not fully leveraging the fitting capability of learnable modules. These methods also struggled with non-rigid deformations. When CNNs were widely used as feature extractors, their potential for cost volumes was further explored. NC-Net [54] first proposed using 4D convolutional layers to learn neighborhood consensus patterns in 4D space, achieving spatially consistent matches between image pairs. However, due to the limitations of the local receptive fields of convolutional layers, NC-Net and follow-ups [55] were constrained by receptive fields, making it difficult to perceive global information during matching. Thanks to the self-attention mechanism [56], transformers had a larger receptive field than CNNs. CATs and their extended methods [57,58] utilized the transformer architecture to calculate and aggregate cost volumes.

### 2.2. Geometry Awareness for Correspondence

Understanding the 3D geometric structure of a class of objects is critical for estimating the semantic correspondences of objects, providing key clues about their spatial structure. Some methods used a single image as a supervisory signal to reconstruct 3D models of objects [59,60,61,62], thereby obtaining correspondences from different viewpoints. These methods generally did not require camera poses or intrinsic and extrinsic parameters. However, a single view only depicts the object from a specific perspective. For structures of the object not captured by the current viewpoint, these methods must rely on prior knowledge or assumptions, such as symmetry, for global prediction. Only images and annotated keypoints are typically available in semantic correspondence tasks, which is especially challenging when dealing with animals in the wild due to scale uncertainty and self-occlusion [63], which often result in distorted 3D model estimates. Consequently, methods based on monocular camera reconstruction to obtain 3D models are difficult to utilize. Using annotated 3D models to render images of objects from novel viewpoints, known as novel view synthesis tasks, combines real and generated data during the training phase to form a consistency loop, allowing the model to perceive matching consistency across viewpoints [63]. Projecting the 3D model onto a densely parameterized canonical surface space [64] creates a set of correspondences at the same location after mapping to the canonical surface space. While the approach can achieve some rotational invariance, it still requires prior information about the 3D model. Ref. [40] introduced a spherical mapping method, mapping features to a category-specific spherical template. The spherical template’s orientation alleviates the problem of distinguishing symmetrical features. This method also introduced an additional viewpoint dataset to explore viewpoint correspondence relationships. However, not all categories fit well onto a spherical template, and this approach performs poorly for categories that are difficult to fit. Multi-sensor fusion methods [65,66] integrate point cloud data from LiDAR or depth sensors with RGB images from RGB sensors, making full use of the complementary strengths of different sensors. The point cloud data contribute depth and geometric information, while the RGB images add detailed appearance and texture. This combination has achieved impressive results in 3D object detection tasks. Our method leverages depth features provided by depth estimation or depth cameras to obtain more geometric structure information, thereby enhancing geometric perception.

The features used in existing methods are typically derived from 2D images, which have limited 3D perception capabilities. Moreover, 3D models reconstructed from single-view images often rely on symmetry assumptions or other priors, making them less reliable. Although integrating multi-sensor data can improve 3D representations, achieving high-quality fusion of multi-sensor data remains a challenge. Additionally, current feature extraction approaches increasingly depend on visual foundation models such as DINO and stable diffusion. However, existing feature fusion strategies have limitations, and the potential of large-scale visual foundation models has not been fully explored.

To address these issues, we first employ a latent depth awareness module to integrate depth feature information with the features from visual foundation models, enhancing the representation capability for objects. Subsequently, a learnable feature fusion module is used to optimize the integration of depth-aware features derived from DINO and SD, fully leveraging the expressive power of both feature types. This approach ultimately improves the accuracy of semantic matching.

## 3. Method

### 3.1. Overview

Given a pair of images $I^{s}$ and $I^{t}$, which include semantic keypoint annotations across the same category, the task of semantic correspondence is to construct the dense correspondence for every pixel between the pair of images.

**Figure 2:**
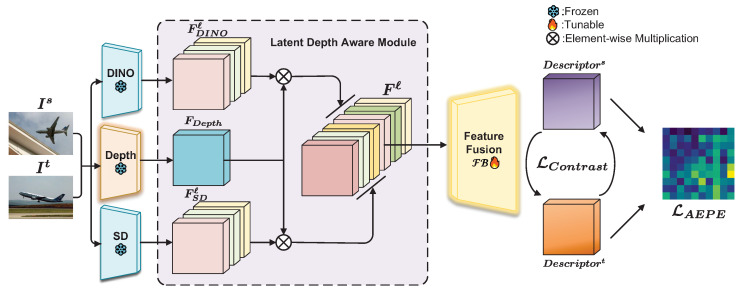
An overview of our method pipeline.

As shown in Figure 2, in the first step, depth information is added to the feature map, enabling it to represent the structural characteristics of the object, resulting in a latent depth awareness representation (Section 3.2). Then, these high-dimensional representations are fused through a feature fusion module guided by learnable weights (Section 3.3). Under the guidance of an improved training objective function (Section 3.4), the learnable weights are optimized to obtain the most effective features for matching.

### 3.2. Latent Depth Awareness Module

**Figure 3:**
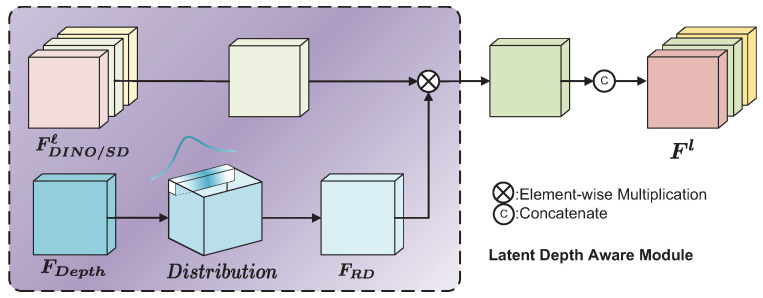
Pipeline of latent depth awareness module.

In semantic correspondence tasks, there is usually no 3D information or camera parameters corresponding to the images, leading to the structural and geometric features of objects being somewhat ignored during feature extraction. Without the camera parameters for every image, it is also very difficult to recover point clouds from depth images because it involves transformations between image coordinate systems, camera coordinate systems, and world coordinates. Some existing methods aim to reconstruct the 3D model of an object from a single view, but due to the lack of sufficient prior information, these methods struggle to obtain accurate and complete 3D models, making it difficult to use them for matching. Additionally, the features learned by SSL models often have difficulty distinguishing between similar parts. To address this problem, inspired by [41,67], we introduce the utilization of depth information to enhance the awareness of structure and geometry. Specifically, as shown in Figure 3, we input the image pairs into a pre-trained depth estimation encoder [68], obtaining the corresponding depth feature map $F_{Depth}$. We then convert this into a discrete probability distribution Distribution. This distribution is fused with the 2D feature map to enhance the model’s utilization of depth information while simultaneously acquiring richer structural and geometric information.

The images are first fed into the pre-trained encoder to generate the feature maps $F_{Depth}$$\in R^{C\times H\times W}$ which contain more structural and geometric information:

(1) $$F_{Depth}=\mathrm{DepthEstimation}\left(I\right)$$

we denote images as *I*$\in R^{C\times H\times W}$, where *C*, *H*, and *W* represent the channels, and the height and width of the image, respectively. Since both the reference image and the target image need to be fed into the module, we do not distinguish between *I* here. Subsequently, we use Softmax(·) to construct the pixel-wise depth distribution, Distribution$\in R^{D\times H\times W}$ from the feature map, where *D* indicates defined discrete depth bins.

(2) $$Distribution=\mathrm{Softmax}(F_{Depth})$$

Here, Distribution$\in R^{D\times H\times W}$ indicates the pixel-wise depth distribution with discrete depth bins $D=\{d_{0},\ldots ,d_{0}+\left(D-1\right)\Delta d\}$. The pixel-wise depth distribution can model the possible depth values at each pixel position, providing the likelihood of different depth values for that position. This also helps to alleviate depth ambiguity at some pixel locations. Based on previous experiences [42], we further utilize the depth distribution Distribution$\in R^{D\times H\times W}$ and the feature map *F*$\in R^{C\times H\times W}$ obtained from the self-supervised vision model to perform an outer product along the channel dimension. This results in depth-aware features *X*$\in R^{D\times C\times H\times W}$ weighted by the depth information. These depth-aware features use the discrete depth distribution to provide depth information for the 2D features. However, storing vectors of the form $R^{D\times C\times H\times W}$ is costly. For the same 2D coordinates (x,y), different discrete depth values and corresponding distribution confidences result in different features, as they share the 2D feature. The main goal of previous methods [41,42] was to generate all possible depth value representations for all pixels to obtain sufficient context information for complex driving scenarios and multi-object situations. However, our method focuses more on the most likely relative depth value for each pixel, using it as part of the object feature. Therefore, we take the discrete interval with the highest confidence as the relative depth representation $F_{RD}$$\in R^{1\times H\times W}$ at that coordinate. Then, we use element-wise multiplication to weight the original *F*, obtaining a latent depth-aware representation, reducing the dimensionality of the depth distribution features.

(3) $$F_{RD}=\mathrm{argmax}\left(Distribution\right)$$

(4) $$F^{\ell }=\mathrm{Conv}\left(F_{DINO/SD}^{\ell }⊗F_{RD}\right)$$

**Figure 4:**
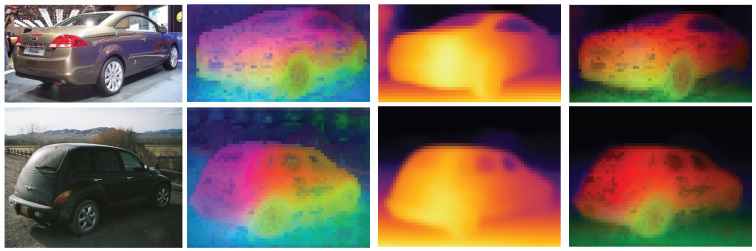
Comparison of PCA from the feature map before and after processing through this module. From left to right: original image, PCA of original feature map, deep feature information, and final result.

Then, the features are further integrated and communicated through the convolution layer of 1×1 to obtain the latent depth awareness representation. Figure 4 shows the results of this process.

### 3.3. Feature Fusion with Learnable Weights

Given feature maps *F*$\in R^{C\times H\times W}$ from generative models and self-supervised vision models following [69], after the latent depth awareness representation is obtained, the multi-layer feature representations need to be fused into a rich descriptor for subsequent cost volume calculation. Existing methods [39] use a fixed weight, applying it separately to the features from the generative model and SSL, then concatenating them. Although this is a simple approach, it can lead to the curse of dimensionality for the descriptor and obscure the principal components of each part of the features. To address this issue, inspired by [38], we note that CNNs [70] still exhibit excellent performance. We designed a feature fusion module based on [70] to merge the features from various layers efficiently.

**Figure 5:**
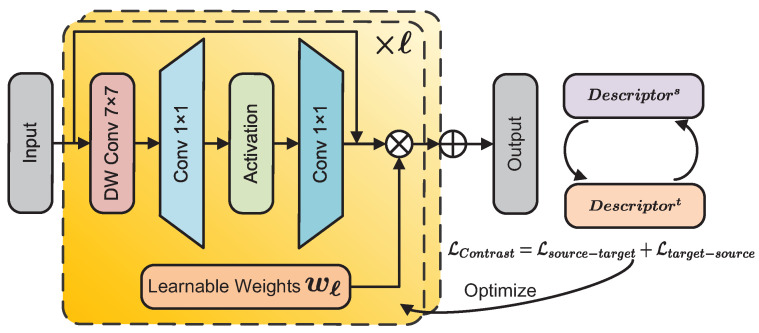
Framework of the feature fusion module.

Figure 5 illustrates the architecture of the proposed learnable feature fusion module, which consists of depthwise convolution, 1×1 convolution layers, and a learnable feature weighting mechanism. First, the module utilizes depthwise convolution (DWConv7×7) to capture local spatial information while reducing computational costs. Then, a 1×1 convolution is used for intra-channel feature aggregation, and a non-linear transformation $GELU\left(\cdot \right)$ is introduced through an activation function, enabling the module to learn more complex representations. Skip connections are employed to alleviate gradient issues and retain certain original information.

To dynamically balance the importance of different feature layers, the module incorporates learnable weights to adaptively emphasize significant features. The fused output features are optimized using a contrastive loss (Section 3.4) to ensure consistency between the source and target features. This fusion module is repeatedly stacked on feature maps at different layers (×ℓ) to gradually optimize feature representation. Subsequently, using the learned weights corresponding to different features, we modulate the features using element-wise multiplication to obtain a descriptor that integrates various features:

(5) $$Descriptor=\sum w_{\ell }\cdot \mathrm{FusionBlock}\left(F^{\ell }\right)$$

where *w* denotes the learnable weight, and *ℓ* represents the number of feature maps. When obtaining diffusion features from different layers, the same time step is used, thereby reducing the impact of the time step on the learnable weights and focusing on the fusion of features from different layers. This feature fusion method better exploits the effective parts of each representation, efficiently integrating them into a single descriptor. It overcomes the curse of dimensionality problem caused by simple concatenation and also alleviates the issue of feature principal component blurring.

### 3.4. Training Objective

Given fused source and target feature maps $F^{s}$, $F^{t}$$\in R^{C\times H\times W}$, respectively, and a set of annotated keypoints, we need to minimize the distances between the same keypoints across different features and maximize the distances between unrelated and different keypoints to distinguish their similarity, which is an insight of contrastive learning. Previous work [38] utilizes a symmetric contrastive loss function from CLIP [28] to achieve this goal. CLIP is originally designed to establish multimodal representations between text and images. It uses InfoNCE [71] to calculate the similarity of embedding positions separately from text-to-image and image-to-text. For previous methods, the contrast between text and image information is directly replaced with the contrast between different images, which is an intuitive substitution. We also adopt this setting to optimize the feature fusion module. However, it is not robust enough under noise interference. Therefore, we apply [72] to modify InfoNCE, reduce the impact of noise on the learning process, optimize the contrastive learning loss function, and enhance the expressiveness of the fused features:

$$\begin{array}{cc} L_{source-target}= & -\frac{1}{N}\left(\frac{\sum_{i=1}^{N}\exp\left(q\cdot \mathrm{Sim}\left({Desc}^{s}\left(i\right),{Desc}^{t}\left(i\right)\right)\right)}{q}\right.\\ \; & \left.-\frac{\sum_{i=1}^{N}\sum_{j=1}^{N}\exp\left(q\cdot \mathrm{Sim}\left({Desc}^{s}\left(i\right),{Desc}^{t}\left(j\right)\right)\right)}{q}\right) \end{array}$$

$$\begin{array}{cc} L_{target-source}= & -\frac{1}{N}\left(\frac{\sum_{i=1}^{N}\exp\left(q\cdot \mathrm{Sim}\left({Desc}^{t}\left(i\right),{Desc}^{s}\left(i\right)\right)\right)}{q}\right.\\ \; & \left.-\frac{\sum_{i=1}^{N}\sum_{j=1}^{N}\exp\left(q\cdot \mathrm{Sim}\left({Desc}^{t}\left(i\right),{Desc}^{s}\left(j\right)\right)\right)}{q}\right) \end{array}$$

(6) $$\begin{array}{c} L_{Contrast}=L_{source-target}+L_{target-source} \end{array}$$

where i,j denote different positions of Descriptor, and robust coefficient *q* is set to 0.5 to balance the robustness of noise and emphasize positive samples. Sim(·) represents the calculation of cosine similarity. Desc indicates Descriptor within the equations to prevent the equations from becoming overly lengthy.

Subsequently, we obtain the similarity matrix between features using cosine similarity. Through the SoftArgmax operation [39], we transform the similarity matrix into a flow field. Assuming that we can obtain the flow field of the ground truth, we apply the Average End Point Error (AEPE) loss, which is the Euclidean distance between flow field vectors, to enforce flow consistency across all pixel points.

$${\hat{u}}_{i}^{t}=\mathrm{SoftArgmax}\left(\sum_{j}\mathrm{Sim}\left(Desc^{s}\left(i\right),Desc^{t}\left(j\right)\right)\right)$$

(7) $$L_{AEPE}=\sum_{i}{∥{\hat{u}}_{i}^{t}-u_{i}^{t}∥}_{2}$$

Here, ${\hat{u}}_{i}^{t}$ indicates the predicted position of the source image on the target image, and $u_{i}^{t}$ represents the ground truth position. We use $L=L_{Contrast}+L_{AEPE}$ as the objective function of the optimization, which can optimize the fusion of features and the key correspondence, respectively.

## 4. Experiments

### 4.1. Implementation Details

Following previous work [39], we modify the size of the original image so that after passing through the two backbone networks, we obtain a series of feature maps *F* of the same size, which are from stable diffusion layers 2, 5, and 8 and DINOv2’s last layer. We train the model using an AdamW optimizer with a $1\times 10^{-3}$ weight decay rate and a cosine annealing learning rate scheduler. Our model is trained on an NVIDIA GeForce RTX 3090 GPU, and other environments are shown in Table 1.

### 4.2. Datasets and Metrics

We evaluate our method on the SPair-71k dataset [73] following existing work [39,58]. SPair-71k is a challenging dataset comprising 1800 images with over 70,000 keypoint pairs to train, test, and validate. Furthermore, we also evaluate our method on a reconstructed AP-10K benchmark [69]. The remade AP-10K benchmark includes 314k keypoint pairs, which is 4× larger than the SPair-71k. The validation and testing image pairs cover three settings: the main intra-species set, the cross-species set, and the cross-family set.

The percentage of correct keypoints (PCK) is a standard practice for matching accuracy and a dimensionless metric, which could be expressed as follows:

(8) $$\mathrm{PCK}=\frac{1}{N}\sum_{i=1}^{N}1\left(\frac{∥{\hat{u}}_{i}-u_{i}∥}{\max(h,w)}\leq \alpha \right)$$

where *N* is the total number of keypoints in the image, ${\hat{u}}_{i}$ denotes the predicted position of the *i*-th keypoint, and $u_{i}$ represents the ground truth position of the of the *i*-th keypoint. *h* and *w* correspond to the height and width of the bounding box surrounding the object, respectively. The term α is a predefined tolerance threshold. The indicator function $1\left(\cdot \right)$ outputs 1 if the normalized distance between the predicted and ground truth keypoints is less than or equal to α, and 0 otherwise. This represents the percentage of predicted keypoints that are within a certain threshold distance from the ground truth. The threshold distance is generally defined by α×max(h,w), where (h,w) denotes the bounding box size of the instances and α represents the tolerance level, usually taking values of {0.01,0.05,0.1}.

### 4.3. Results

Based on the experimental results of different methods on the SPair-71k test set shown in Table 2, it can be seen that our method achieves outstanding performance in most categories and overall results (+7.3%). Table 3 shows the experimental results obtained with different tolerance levels α on the SPair-71k and reconstructed AP-10K datasets. Our method performs well across α∈{0.01,0.05,0.1}, surpassing previous methods (+9% average). The performance of the model remains relatively stable across various sets in the AP-10K dataset.

### 4.4. Discussion

Table 4 presents the matching accuracy (PCK@0.1) and the average inference time per image pair for three existing methods and our method on the SPair-71K dataset, all tested under the same hardware conditions described in Table 1.

**Figure 6:**
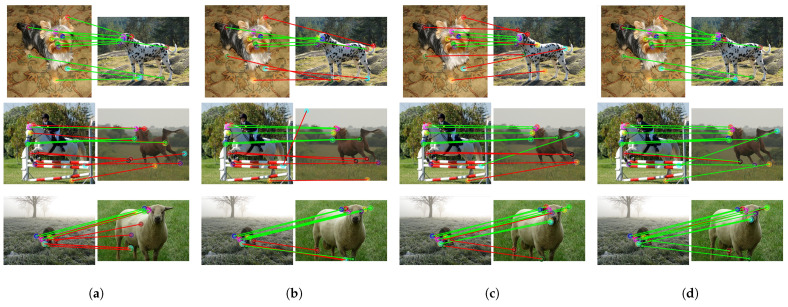
Qualitative comparison of dog, horse and sheep categories. Green lines represent correct matches, and red is incorrect. (**a**) Result of CATs++ [58], (**b**) result of DHF [38], (**c**) result of SD+DINO [39], (**d**) our result. Green lines represent correct matches, and red is incorrect.

The CATS++ method [58], which performs cost aggregation using an improved attention architecture, can achieve relatively accurate matching results in simple environments, as shown in Figure 6. However, the matching results are unstable, and in more challenging environments, it struggles to obtain correct matches for objects with significant variations. Although the accuracy is slightly lower, CATS++ has the highest computational efficiency due to its backbone feature extraction network based on ResNet with a transformer as an auxiliary. During inference, it only takes 78.8 ms to match a pair of images, as shown in Table 4.

Compared to CATS++, our method provides more stable features, but this comes at the cost of a significantly longer inference time. Nevertheless, the use of robust and effective features results in more accurate matching, indicating a trade-off between inference speed and accuracy.

The DHF [38] method integrates SD features at multiple scales and time steps as descriptors. The SD features can capture certain spatial information. However, compared to our method, DHF lacks the self-supervised features derived from DINO, which leads to slightly poorer performance for some parts of objects, such as the legs of animals or the wheels of cars. Regarding inference time, due to the extraction and fusion of multiple SD feature layers, the inference time is longer than ours.

When both SD and DINO features are used [39], the inclusion of DINO features significantly improves the matching accuracy by introducing richer semantic information. However, merely using PCA to fuse features will lead to feature blurring. For the matching results generated by our model, the inclusion of the latent depth awareness module naturally incorporates depth information into both features, enhancing the understanding of object structures. Additionally, the learnable feature fusion module, which is trained to fully adjust and utilize both types of features, maximizes the potential of each component, leading to better matching results. In terms of inference time, due to the SD model, the inference time is similar to other methods using the SD model. Our method takes 361 ms longer than SD+DINO to complete the matching for a pair of images.

**Figure 7:**
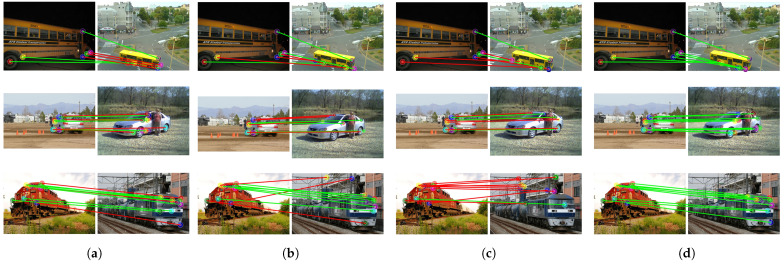
Qualitative comparison of bus, car, and train categories. Green lines represent correct matches, and red is incorrect. (**a**) Result of CATs++ [58], (**b**) result of DHF [38], (**c**) result of SD+DINO [39], (**d**) our result. Green lines represent correct matches, and red is incorrect.

**Figure 8:**
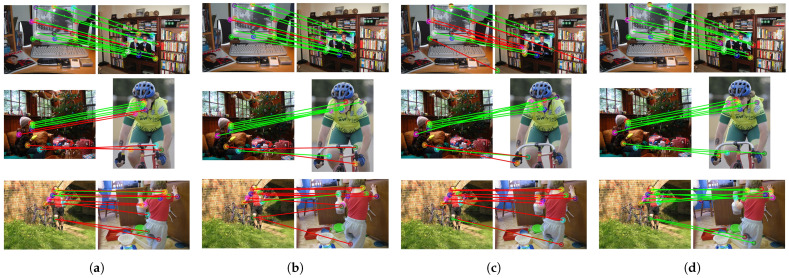
Qualitative comparison of person and TV categories. Green lines represent correct matches, and red is incorrect. (**a**) Result of CATs++ [58], (**b**) result of DHF [38], (**c**) result of SD+DINO [39], (**d**) our result. Green lines represent correct matches, and red is incorrect.

Based on the visual results of the corresponding matches shown in Figure 6, Figure 7 and Figure 8, the first set of comparison results are shown in Figure 6, including a dog, horse, and sheep. In previous methods, the matching performance for the head was generally good, but the matching for the limbs was not satisfactory, often resulting in all keypoints of different limbs being matched to the same limb. Our method, by incorporating relative depth information, enables the model to understand the front–back relationship in the feature map, thereby alleviating the matching issues for the limbs seen in previous methods. The second set addresses the matching of vehicles, including buses, cars, and trains, shown in Figure 7. Even with significant variations in vehicle posture, the matching can still be performed correctly, which is closely related to the efficient and robust representations provided by SSL and SD. Additionally, the feature fusion module we applied enhances the utilization of feature representations. Furthermore, depth information increases the amount of information for parts such as the front and rear wheels.

Based on the qualitative results, it can be seen that while the previous methods are confused by depth ambiguity, our method can correctly find the correspondences and obtain the correct keypoint positions in the target image. As mentioned in Section 3.2, SSL models learn more invariant features. Invariant features refer to characteristics that remain consistent and unaffected when facing various input variations. In machine learning and deep learning, especially in supervised learning scenarios, models learn such features to enhance robustness against changes in data samples or distributions. However, when handling subtle differences such as the distinction between “the front and rear wheels of a car”, the nature of invariant features—making the model insensitive to certain irrelevant transformations and perturbations—limits its ability to capture such fine details. Our method incorporates a depth awareness module, adding more structural information of the object into the features, which gives the original features sufficient depth information weighting. This alleviates matching problems in scenarios with depth ambiguity.

### 4.5. Ablation Studies

Table 5 shows the experimental results between the baseline method [39] and our proposed method. We started from the baseline method on the SPair-71k dataset and gradually added our method. First, we replaced the fixed-weight feature fusion with our feature fusion module. The results show that the learnable weight mode and the method of aligning features to a similar space significantly improved feature fusion, with PCK@0.1 increasing by approximately seven points. There were also significant improvements in PCK@0.01 and PCK@0.05, which have lower tolerance levels.

Therefore, when fusing features, mapping the features to a similar latent space and then using learnable weights for fusion can better utilize features from each layer, making a greater contribution and impact on the final descriptor.

Next, before feature fusion, we use depth information to weigh the features from each layer, attaching more structural information from the depth data to the features from each layer to obtain the latent depth representation. Then, we perform feature fusion. The results show that including depth information improves the PCK metric at different tolerance levels. This indicates that adding depth information allows the model to understand scenes with depth ambiguities better, enhancing the perception of object structures. It can also attach different depths to the invariant features of SSL, making them easier to distinguish.

### 4.6. Limitations

**Figure 9:**
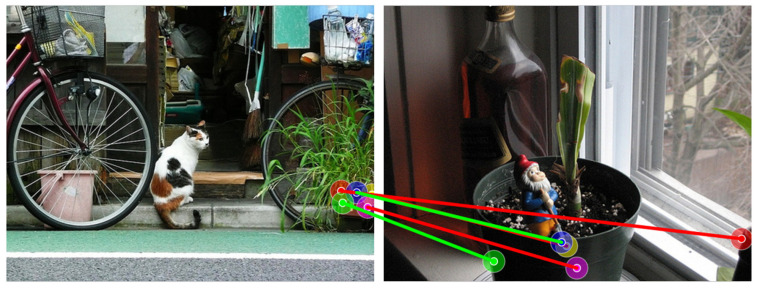
The limitation of scale differences.

Both the previous methods and our method require the extraction of DINO and SD feature maps in advance for training. If we do not extract the feature maps in advance, our current equipment’s (Table 1) training and inference time will increase by several times. This indicates that large-scale vision models like DINO and SD have huge computational demands, even with good representation capabilities. This also means that our method will be difficult to run in real time. In the future, accelerating or distilling [76] large vision models will remain an interesting problem. Additionally, handling images with significant scale differences remains challenging, as illustrated in Figure 9.

## 5. Conclusions

We proposed a latent depth awareness module and a feature fusion module with learnable weights to activate the model’s perception of geometric structures and better integrate self-supervised learning and generative model features. The key insight is that the depth map from depth estimation or depth sensors contains rich structural information, and learnable weights can better allocate the fusion of various feature maps. Our method models the pixel-wise depth distribution of depth features to obtain depth representation, performing depth weighting to integrate depth information into the features. By leveraging depth information, our approach not only improves geometric understanding but also demonstrates the potential of deep learning to activate the fusion of multi-sensor data. Experiments show that our method significantly outperforms previous methods by seven points on the SPair-71K dataset and ten points PCK@0.1 on the AP-10K dataset.

Our method has promising application potential in fields such as style/appearance transfer, robot manipulation, and zero-shot pose estimation. The incorporation of semantic matching as a supplement to image matching plays a crucial role in improving the accuracy of these downstream tasks.

Despite the performance improvements achieved by our method, certain limitations remain. First, the effectiveness of our approach partially depends on the accuracy of depth information. When reliable and accurate depth information cannot be obtained, the performance of our method may be compromised. Second, the introduction of the depth awareness module and the utilization of SD and DINO increase computational complexity, limiting the real-time applicability of our method.

To address these issues, future work will focus on optimizing computational efficiency and exploring multimodal fusion strategies, such as incorporating LiDAR data, to enhance the model’s performance in complex scenarios. Additionally, future research will extend the model to handle multi-category and complex scene matching tasks, and we will conduct evaluations on larger-scale datasets to further improve the robustness and generalization of the model.

## Figures and Tables

**Table 1:** Experimental environment configuration.

Hardware and Software	Name
Operating system	Ubuntu 20.04.5 LTS (Canonical, London, UK)
CPU	Intel Core i5-12400 4.0GHz (Intel, Santa Clara, CA, USA)
GPU	NVIDIA GeForce RTX 3090 (NVIDIA, Santa Clara, CA, USA)
RAM	48GB
Python version	3.9.19 (Python Software Foundation, Wilmington, DE, USA)
Dependency	Pytorch 1.13.1/ torchvision 0.14.1/CUDA 11.6
	(Meta Platforms, Menlo Park, CA, USA; NVIDIA, Santa Clara, CA, USA)

**Table 2:** Evaluation results on testing sets of SPair-71k dataset. Per-class and average PCK at 0.1 tolerance level on the testing set of SPair-71K. We report per-image PCK metrics. The bolded data represents the highest accuracy in that column.

Methods	Airplane	Bicycle	Bird	Boat	Bottle	Bus	Car	Cat	Chair	Cow	Dog	Horse	Motorbike	Person	Potted plant	Sheep	Train	TV	All
SCOT [53]	34.9	20.7	63.8	21.1	43.5	27.3	21.3	63.1	20.0	42.9	42.5	31.1	29.8	35.0	27.7	24.4	48.4	40.8	35.6
PMNC [74]	54.1	35.9	74.9	36.5	42.1	48.8	40.0	72.6	21.1	67.6	58.1	50.5	40.1	54.1	43.3	35.7	74.5	59.9	50.4
SCorrSAN [75]	57.1	40.3	78.3	38.1	51.8	57.8	47.1	67.9	25.2	71.3	63.9	49.3	45.3	49.8	48.8	40.3	77.7	69.7	55.3
CATs++ [58]	60.6	46.9	82.5	41.6	56.8	64.9	50.4	72.8	29.2	75.8	65.4	62.5	50.9	56.1	54.8	48.2	80.9	74.9	59.8
DHF [38]	74.0	61.0	87.2	40.7	47.8	70.0	74.4	80.9	38.5	76.1	60.9	66.8	66.6	70.3	58.0	54.3	87.4	60.3	64.9
SD+DINO [39]	81.2	66.9	91.6	61.4	57.4	85.3	83.1	**90.8**	54.5	88.5	75.1	80.2	71.9	77.9	60.7	68.9	92.4	65.8	74.6
**Ours**	**83.7**	**72.3**	**96.0**	**67.0**	**64.2**	**93.1**	**86.8**	90.5	**67.9**	**91.8**	**83.7**	**84.7**	**78.7**	**85.9**	**80.1**	**77.1**	**95.8**	**81.1**	**81.9**

**Table 3:** Evaluation results on SPair-71k and reconstructed AP-10K datasets at various PCK levels. We report the performance on the intra-species, cross-species, and cross-family testing sets of the AP-10K dataset. We report the per-image PCK results.The bolded data represents the highest accuracy in that column.

	SPair-71k			AP-10K Intra-Species			AP-10K Cross-Species			AP-10K Cross-Family		
**Methods**	**0.01**	**0.05**	**0.10**	**0.01**	**0.05**	**0.10**	**0.01**	**0.05**	**0.10**	**0.01**	**0.05**	**0.10**
CATs++ [58]	4.3	40.7	59.8	-	-	-	-	-	-	-	-	-
DHF [38]	8.7	50.2	64.9	8.0	45.8	62.7	6.8	42.4	60.0	5.0	32.7	47.8
SD+DINO [39]	9.6	57.7	74.6	9.9	57.0	77.0	8.8	53.9	74.0	6.9	46.2	65.8
**Ours**	**18.5**	**70.3**	**81.9**	**21.8**	**72.6**	**86.2**	**21.6**	**70.3**	**85.4**	**19.4**	**64.6**	**79.7**

**Table 4:** Quantitative comparison of inference time and PCK@0.1 accuracy. The bolded data represents the best result in that column.

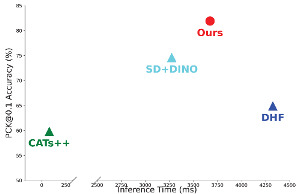		
Methods	Inference Time (ms)	PCK@0.1(%)
CATs++	**78.8**	59.8
DHF	4325.8	64.9
SD+DINO	3275.2	74.6
Ours	3637.1	**81.9**

**Table 5:** Ablation studies on testing sets of SPair-71k. The “+” symbol indicates the incremental addition of proposed module.

		SPair-71k		
	**Model Ablations**	**0.01**	**0.05**	**0.10**
	Baseline	9.6	57.7	74.6
**+**	Feature fusion with learnable weights	17.8	69.4	80.7
**+**	Latent depth representation	18.5	70.3	81.9

## Data Availability

The data are contained within the article.

## Abbreviations

Abbreviation	Full Term
SLAM	Simultaneous Localization And Mapping
PCK	Percentage of Correct Keypoints
CNN	Convolutional Neural Networks
SSL	Self-Supervised Learning
SD	Stable Diffusion
PCA	Principal Component Analysis
